# Supplementary material for: Integrative Analysis Identifies TCIRG1 as a Potential Prognostic and Immunotherapy-Relevant Biomarker Associated with Malignant Cell Migration in Clear Cell Renal Cell Carcinoma
Source: Cancers (Basel). 2022 Sep 21;14(19):4583. doi: 10.3390/cancers14194583 (PMC9558535; doi:10.3390/cancers14194583)
Supplement: Supplementary file 1 [file cancers-14-04583-s001.zip › cancers-1880543-supplementary.pdf]

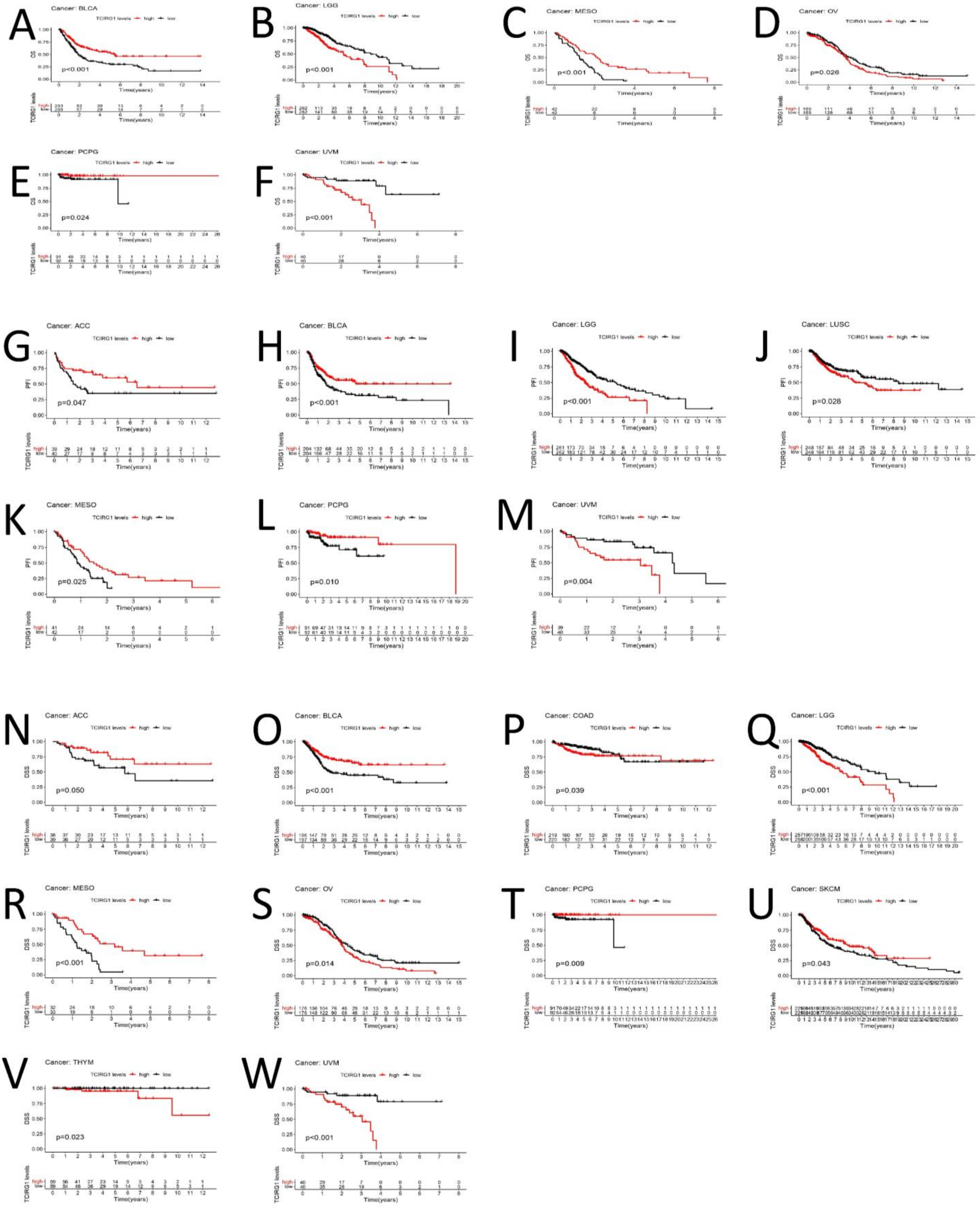

Figure S1: KM survival analysis of the association between TCIRG1 expression and OS (A–F), PF (G–M), and DSS (N–W).

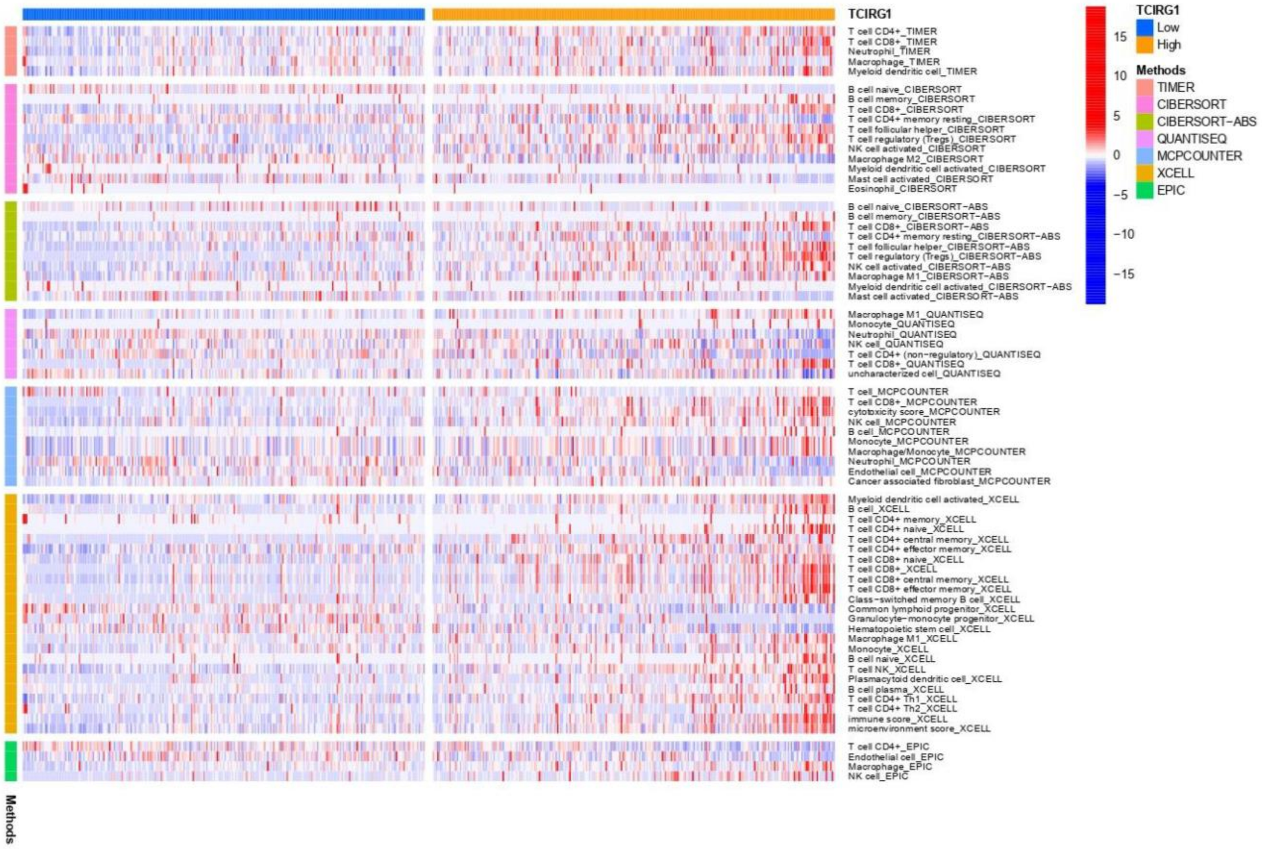

Figure S2: The relationship between TCIRG1 and immune cells in renal clear cell carcinoma tissue is revealed by several algorithms.

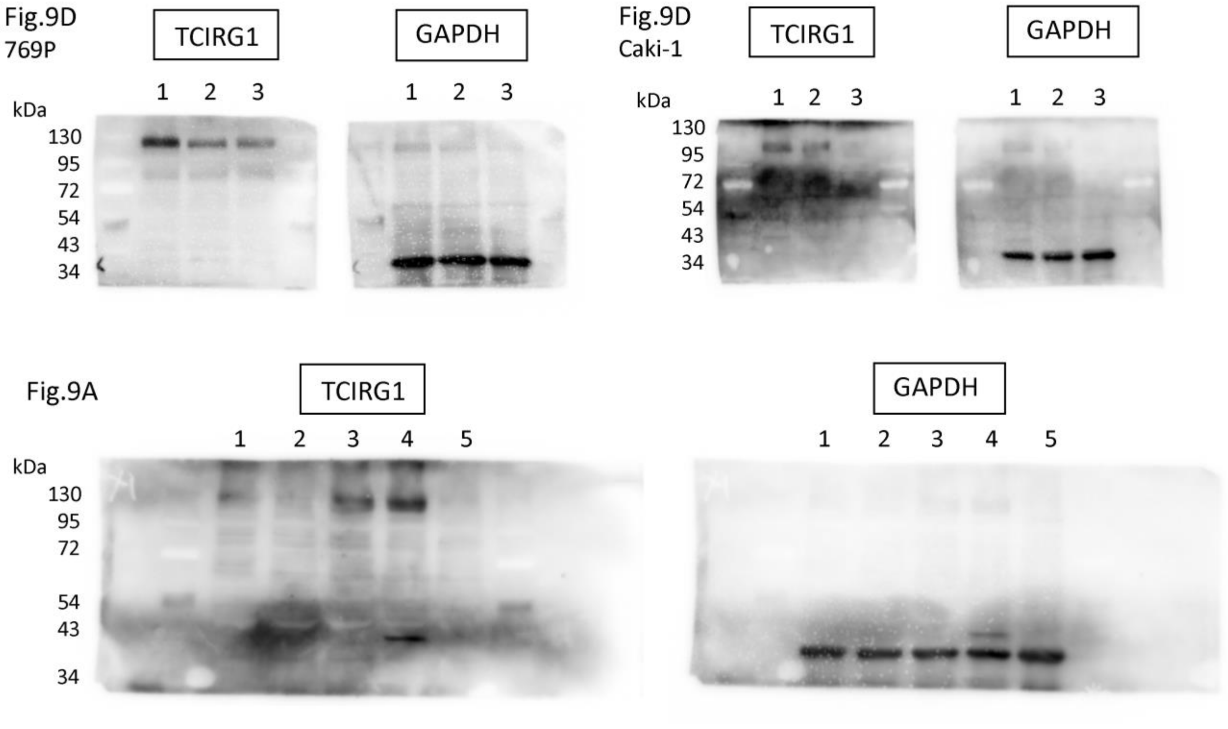

Figure S3: uncropped original western blots of Figure 9.
